# The *CLIMATE schools combined* study: a cluster randomised controlled trial of a universal Internet-based prevention program for youth substance misuse, depression and anxiety

**DOI:** 10.1186/1471-244X-14-32

**Published:** 2014-02-05

**Authors:** Maree Teesson, Nicola C Newton, Tim Slade, Cath Chapman, Steve Allsop, Leanne Hides, Nyanda McBride, Louise Mewton, Zoe Tonks, Louise Birrell, Louise Brownhill, Gavin Andrews

**Affiliations:** 1 NHMRC Centre for Research Excellence in Mental Health and Substance Use, National Drug and Alcohol Research Centre, University of New South Wales, 22-32 King Street, Randwick, NSW 2052, Australia; 2 National Drug Research Institute, Curtin University, Perth, Australia; 3 Queensland University of Technology, Brisbane, Australia; 4 Clinical Research Unit for Anxiety and Depression, St Vincent’s Hospital, University of New South Wales, Sydney, Australia

**Keywords:** Prevention, School, Internet, Universal, Randomised controlled trial, Mental health, Depression, Anxiety, Substance use

## Abstract

**Background:**

Anxiety, depressive and substance use disorders account for three quarters of the disability attributed to mental disorders and frequently co-occur. While programs for the prevention and reduction of symptoms associated with (i) substance use and (ii) mental health disorders exist, research is yet to determine if a combined approach is more effective. This paper describes the study protocol of a cluster randomised controlled trial to evaluate the effectiveness of the *CLIMATE Schools Combined* intervention, a universal approach to preventing substance use and mental health problems among adolescents.

**Methods/design:**

Participants will consist of approximately 8400 students aged 13 to 14-years-old from 84 secondary schools in New South Wales, Western Australia and Queensland, Australia. The schools will be cluster randomised to one of four groups; (i) *CLIMATE Schools Combined* intervention; (ii) *CLIMATE Schools - Substance Use; (iii) CLIMATE Schools - Mental Health*, or (iv) Control (Health and Physical Education as usual).

The primary outcomes of the trial will be the uptake and harmful use of alcohol and other drugs, mental health symptomatology and anxiety, depression and substance use knowledge. Secondary outcomes include substance use related harms, self-efficacy to resist peer pressure, general disability, and truancy. The link between personality and substance use will also be examined.

**Discussion:**

Compared to students who receive the universal *CLIMATE Schools - Substance Use*, or *CLIMATE Schools - Mental Health* or the Control condition (who received usual Health and Physical Education), we expect students who receive the *CLIMATE Schools Combined* intervention to show greater delays to the initiation of substance use, reductions in substance use and mental health symptoms, and increased substance use and mental health knowledge.

**Trial registration:**

This trial is registered with the Australian and New Zealand Clinical Trials registry, ACTRN12613000723785.

## Background

Substance use, anxiety and depressive disorders account for three quarters of the disability attributed to mental disorders [1]. The peak of this disability occurs in those aged 15-24 years old and corresponds with the typical period of onset of these problems. Furthermore, substance use, anxiety and depressive disorders frequently co-occur, share common risk factors and interact [2]. To reduce the occurrence and cost of such disorders, it is imperative that preventative interventions begin early, before patterns of anxiety, depressive and substance use symptoms are established and begin to cause disability, as well as vocational, educational and social harm [3-5].

An array of substance use prevention programs have existed for some time [6-11]. However, the majority of programs have shown minimal effects in reducing substance use and related harms [12-14], and some have even reported detrimental effects [15]. It has been suggested that the most common factors which interfere with effectiveness are; the focus on abstinence‒based outcomes [16,17], and implementation failure [18-20].

Problems with the implementation and dissemination of drug prevention programs are well-recognised in the drug prevention field [18,21-26]. First, programs with proven efficacy are often not widely used [27]. Botvin & Griffin [27] propose that this is due to the lack of compatibility of evidence-based programs designed by researchers with school curriculums. As such, schools often opt for commercial programs that are appealing, yet lack supporting scientific evidence. In addition, schools often do not have sufficient motivation or resources to adequately train educators to teach such program [7,28]. Specifically, a US study found only 17 per cent of schools reported training teachers in substance use prevention programs [28]. This is problematic as teachers may adapt or remove essential program components and inadvertently detract from the effectiveness of the program [29]. Drug prevention programs with the most success in increasing alcohol and other drug related knowledge, decreasing pro-drug attitudes and decreasing drug use behaviour, are those containing harm-minimisation goals and interactive delivery techniques, which are correctly implemented [30-34]. For example, a meta-analysis by Tobler et al. [35] found schools in the United States that implemented both the content and delivery methods of the prevention programs had the largest effect sizes. Nevertheless, a study conducted by Ennett et al. [19] found only 14 per cent of schools implemented both the content and delivery methods of drug prevention programs [35].

The effectiveness of prevention programs for anxiety and depressive symptoms is also contentious. For example, ‘targeted’ prevention programs for young people at high risk of a depressive disorder, have been shown to be effective over the long term, while universal school-based programs for the prevention of depression in young people have not consistently demonstrated positive effects [36-38]. Here, it has been found that whilst some universal prevention programs are effective for reducing symptoms in the short term, there is currently no evidence for long term effects [36].

Programs to prevent and reduce anxiety symptoms have also yielded mixed results. A targeted intervention, comparing a 10 session cognitive-behavioural therapy (CBT) intervention with a waitlist control among 128 (out of 1,786 screened) highly anxious 14 year olds, found the CBT group had half the risk of meeting criteria for an anxiety disorder compared to the control group (20% vs. 39%) at two-years follow up [39]. An adaption of this CBT intervention into a school-based ‘universal’ prevention program, called FRIENDS, appears to be the only school based prevention program that is effective for both anxiety and depression [40]. However, the reliance of this program on input from trained professionals, such as clinical psychologists and school counsellors, makes the program difficult and costly to implement as part of routine practice within schools.

The results of a randomised controlled trial (RCT) of a ‘targeted’ version of the FRIENDS program, delivered by trained school staff, as opposed to clinicians, were disappointing in that the intervention group showed no gains [41]. The majority of schools also failed to continue implementing the program after the four year trial. These findings underscore the need for more innovative school-based programs that are less time and resource intensive, as well as less reliant on outside monitoring and support for compliance and implementation [38]. Other prevention programs aimed at preventing and reducing anxiety and depressive symptoms among adolescents include MoodGYM and the Aussie Optimism Program [42,43]. Trials of the Aussie Optimism Program have yielded mixed effects in anxiety and depressive symptoms [43]. In contrast, the MoodGYM (YouthMood) program yielded significant differences in anxiety symptoms in adolescents up to 17 years old, however the benefits for depressive symptoms were less strong [42]. Importantly, only MoodGYM delivers their course online, thereby supporting the feasibility of Internet-based delivery [38].

Taken together, the aforementioned programs show promising results and highlight the importance of implementation fidelity for the development of more effective programs to prevent and reduce substance use and symptoms of anxiety and depression. Another key point is that current programs are designed to target either substance use or mental health disorders, despite research indicating that these disorders co-occur [2]. As such, a combined approach to prevent and reduce symptoms associated with these common disorders may result in greater symptom reduction, be more cost effective and feasible compared to separate mental health and substance use interventions.

The current study seeks to evaluate an innovative combined approach to prevent and decrease substance use and mental health symptoms in Australian adolescents. The proposed universal program, known as the *CLIMATE Schools Combined (CSC)* program*,* will combine the universal *CLIMATE Schools - Substance Use* program and the universal *CLIMATE Schools - Mental Health* program. This program will utilise Internet-based delivery in schools, which has been identified as an effective method to address implementation issues [38,44]

### The *CLIMATE Schools Combined (CSC)* program

The *CLIMATE Schools Combined* program consists of both the *CLIMATE Schools - Substance Use* program and the *CLIMATE Schools - Mental Health* program. All *CLIMATE Schools* programs include both computer-based and manualised classroom activities. The computer-based component is completed individually via the Internet, where students are engaged through contemporary cartoon storylines that impart information about substance use, and anxiety and depressive symptoms. Students are provided with confidential login details to access the *CLIMATE Schools* website. The classroom activities are delivered by the teacher and aim to reinforce the learning outcomes outlined in the cartoons and allow interactive communication between students. Teachers are provided with access to all program materials including an outline of activities, implementation guidelines, links to the education syllabus and summaries for each lesson. Implementation issues are also addressed, as the program is designed to link with the Australian National Curriculum and Australian state-based Health and Physical Education syllabuses. The program is also facilitated by the Internet to maximise efficient, complete and consistent delivery, as well as ensure high implementation fidelity.

### The universal *CLIMATE Schools - Substance Use* program

The universal *CLIMATE Schools - Substance Use* program aims to reduce the use of alcohol and cannabis, Australia’s most commonly used licit (excluding caffeine) and illicit drugs [45]. The program is designed for students aged 13-14 years old, when significant exposure to alcohol and drug use occurs. The program is based on the harm-minimisation approach to prevention, as there is a growing body of evidence to suggest that this approach may be more effective than the abstinence only approach [44,46].

The *CLIMATE Schools - Substance Use* program consists of twelve 40-minute lessons. The first six lessons focus specifically on alcohol and are delivered approximately six months prior to the remaining six lessons, which focus on cannabis.

The efficacy of the *CLIMATE Schools - Substance Use* program has been established using a cluster RCT in 10 schools in Sydney, Australia (n = 764) [47,48]. Results of the trial demonstrated that students in the intervention group made significant improvements in alcohol and cannabis knowledge at the end of the program and at 6 and 12 months post-intervention, in comparison to the control group (where students received drug education as usual). The intervention group also showed a reduction in frequency of cannabis use at the 6-month follow-up, a reduction in average weekly alcohol consumption at the 6 and 12 month follow-up, and a reduction in frequency of drinking to excess 12 months following the intervention. In addition, students participating in the *CLIMATE Schools* program, exhibited high follow-up rates, thereby suggesting that the Internet is an effective method of delivery. Importantly, students and teachers rated the program as acceptable and enjoyable. Specifically, 100% of teachers who implemented this program in their classroom rated it as superior to other substance use prevention programs, and over 90% of students reported information delivered in this format was easy to learn and would like more school subjects to be taught using this method.

### The universal *CLIMATE Schools - Mental Health* program

The universal *CLIMATE Schools - Mental Health* program aims to prevent and reduce anxiety and depressive symptoms in young people. The program is designed for adolescents aged 14-15 years old, as research demonstrates that programs are effective in reducing these symptoms when delivered at this time [37,38]. This program is based on cognitive-behavioural principles and incorporates skill acquisition; psycho education, management of psychological symptoms, cognitive symptoms, behaviour and additional skills specific to anxiety and depression. There are six 40-minute lessons, which are also curriculum-based.

### Updating the *CLIMATE Schools - Mental Health* program

The *CLIMATE Schools- Mental Health* program is currently being updated and is based on two modules from the online school education program provided by the ‘This Way Up’ clinic, titled ‘*Overcoming Anxiety’* and ‘*Combating Depression’*. Originally six lessons each, the two modules have been condensed into the six lesson *CLIMATE Schools - Mental Health* program, in order to reduce content overlap and decrease time demands for participating schools. In revising the program, an experienced clinical psychologist amended the script of the online cartoon component, and illustrations were updated to reflect current youth trends and to maintain consistency with the *CLIMATE Schools - Substance Use* program. Focus groups were conducted with approximately 30 students from two independent schools in Sydney, Australia to ensure that the modifications accurately reflected realistic scenarios and language of Year 9 students. Once the *CLIMATE Schools - Mental Health* program has been modified, a number of health and educational professionals will be approached to comment on the program’s clinical and educational validity, and content will be revised accordingly.

### Objectives of the CSC study

The primary objective of the CSC Study is to evaluate an innovative Internet-based prevention program for the prevention and reduction of substance use, anxiety and depressive symptoms, by combining the universal *CLIMATE Schools - Substance Use* program and the *CLIMATE Schools - Mental Health* program.

It is hypothesised that the CSC program will be more effective than; (1) school-based Health and Physical Education as usual, (2) the *CLIMATE Schools - Substance Use* program, and (3) the *CLIMATE Schools - Mental Health* program in relation to the following outcomes:

1) reducing the use and harmful use of alcohol and cannabis

2) reducing overall levels of anxiety and depression

3) increasing knowledge related to alcohol, cannabis anxiety and depression.

Secondary aims include examining the effects of the interventions on alcohol use related harms, self-efficacy to resist peer pressure, general disability, and truancy. The link between personality and substance use, as well as mental health will also be examined.

## Method/design

In 2012 the CSC project was funded as part of a National competitive funding round.^1^ Ethical approval has been granted from the UNSW Human Research Ethics Committee (HC13073) and from each school.

### Study design

To establish the effectiveness of the integrated CSC intervention, a cluster RCT will be conducted in 84 Australian secondary schools between 2014 and 2016. Cluster randomisation will be employed to avoid contamination of the control group with the intervention group through student communication. Participating schools will be randomly allocated to one of four groups; (1) the *CLIMATE Schools - Combined* condition (CSC), (2) the *CLIMATE Schools* - *Substance Use* condition (CS-SU), (3) the *CLIMATE Schools - Mental Health* condition (CS-MH), or (4) the Control condition (CO). See Table 1 for a graphical display of the experimental design.

**Table 1:** Experimental design

		**Substance use**	
		+	-
**Mental health**	+	*CS-C	*CS-MH
	-	*CS-SU	*CO

Key:

*CO = Control condition.

*CS-MH = Climate Schools Mental Health condition.

*CS-SU = Climate Schools Substance Use condition.

*CS-C = Climate Schools Combined condition.

### Sample size calculations

To account for cluster randomisation, sample size calculations were based on recent sample size requirements developed by Heo & Leon [49]. to detect intervention by time interactions in longitudinal cluster randomised clinical trials. The CSC trial will be powered to detect differences in the overall sample as well as between samples from each of four groups within the three States of NSW, QLD and WA. To allow for comparisons within each State, six schools (with at least 100 students) in each of the four intervention groups are required per state, giving a total of 24 schools per State (at least 2400 students). This achieves 80 per cent power to detect a standardized between-group mean difference of 0.15 (p = 0.05) in outcomes at the end of the trial with seven measurement occasions. An effect size of 0.15 is comparable to previous school-based trials of universal mental health and substance use prevention programs and would have substantial benefits on a population level based on recent economic modelling [50]. To account for school dropouts during the trial, which we expect to be approximately 10 per cent, we aim to recruit at least 28 schools in each State (at least 2800 students). This will result in an overall sample of 8400 students from 84 schools at baseline to test the efficacy of the CSC intervention in comparison to the other groups. Based on previous research using the *CLIMATE Schools* programs, school participation rates for similar trials were approximately 30 per cent [48,51]. Therefore, to recruit a total of 84 schools, approximately 270 schools will be approached to participate.

### Procedure

#### Recruitment of schools and randomisation

**Figure 1:**
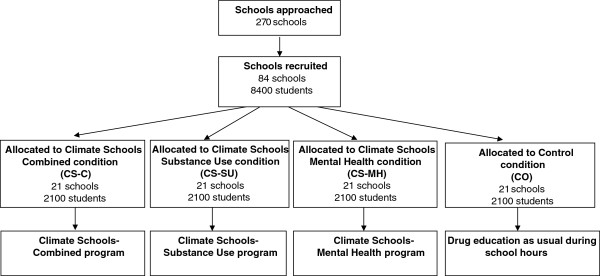
Flow chart of anticipated overall allocation of schools to the intervention groups.

The recruitment, inclusion, and randomisation of the participants (schools and students) will commence in September 2013. A total of 270 schools will be selected from a list of all public, independent and catholic schools in NSW, QLD and WA. The Principals of these schools will be sent a letter outlining the aims of the research, school requirements, time-frames, details of the randomisation procedure and an invitation for their school to participate. School Principals will then be followed up with a further email and phone call to invite their school to participate and explain the study requirements. Following school consent, randomisation will occur using the *Ralloc* function in Stata, and schools will be allocated to one of the four intervention groups shown in Figure 1.

Information and consent forms will be sent home to parents/guardians of all Year 8 students (Year 9 for QLD) aged between 13-14 years, at participating schools. Only students who receive parental consent and give consent themselves will be eligible to be involved in the study.

#### ***Interventions***

Following the baseline assessment, students in the CS-SU group will receive the *CLIMATE Schools* - *Substance Use* program during Year 8 (Year 9 in QLD), while aged 13-14 years old. Those students allocated to the CS-MH group will receive the *CLIMATE Schools - Mental Health* program during Year 9 (Year 10 in QLD), when aged 14-15 years old. Students in the CSC group will receive both the *CLIMATE Schools - Substance Use* and *CLIMATE Schools - Mental Health* programs over Year 8 and 9 (Year 9 and 10 in QLD).

### Control group

Students randomised to the *Control* group will receive their usual Health and Physical Education classes (including lessons on drugs, alcohol, and mental health) over the three years of the intervention period. Control schools will be asked to record any substance use and/or mental health education they deliver during the year, including how many lessons and the format of such lessons. Control schools will be offered the use of the CSC program following completion of the study. See Figure 1 for intervention breakdown by group.

#### ***Assessment occasions***

Regardless of the condition to which schools are assigned, all students will be assessed via a self-report questionnaire at; baseline, immediately pre and post each *CLIMATE Schools* program (i.e., *Substance Use* and *Mental Health* programs) and 18, 24 and 30 months after baseline. Schools will have the option to complete the assessments either online or by paper and pencil. Each student will be assigned a unique username and password to login to the study website and all survey data obtained is strictly confidential. Table 2 displays the anticipated CSC study assessment times.

**Table 2:** Climate Schools Combined (CSC) study timeline

	**Survey 1**	***Climate Schools- Substance Use (module 1)***	***Climate Schools – Substance Use (module 2)***	**Survey 2**	**Survey 3**	***Climate Schools-Mental Health***	**Survey 4**	**Survey 5**	**Survey 6**	**Survey 7**
**Time**	**Term 1**	**Term 1**	**Term 3**	**Term 3**	**Term 1**	**Term 1**	**Term 1**	**Term 3**	**Term 1**	**Term 3**
	**2014**	**2014**	**2014**	**2014**	**2015**	**2015**	**2015**	**2015**	**2016**	**2016**
CS-C*	✓	X	X	✓	✓	❖	✓	✓	✓	✓
CS-SU*	✓	X	X	✓	✓		✓	✓	✓	✓
CS-MH*	✓			✓	✓	❖	✓	✓	✓	✓
CO*	✓			✓	✓		✓	✓	✓	✓

Key:

*CS-C = Climate Schools Combined group.

*CS-SU = Climate Schools - Substance Use intervention only group.

*CS-MH = Climate Schools - Mental Health intervention only group.

*CO = Control group.

✓ = Measurement occasion.

Χ = Climate Schools - Substance Use 12 lesson intervention.

❖ = Climate Schools - Mental Health 6 lesson intervention.

Survey time points:

Survey 1: Baseline.

Survey 2: Post-Substance Use intervention.

Survey 3: Pre-Mental Health intervention.

Survey 4: Post-Mental Health intervention.

Survey 5: 6-month follow-up.

Survey 6: 12-month follow-up.

Survey 7: 18-month follow-up.

### Measures

Demographic data including gender, age, country of birth, academic performance, and truancy rates will be obtained to determine baseline equivalence of the groups.

#### ***Alcohol and other substance use***

Students will be asked to rate the frequency and quantity of their alcohol consumption in standard drinks, frequency of drinking to excess (defined as having more than five standard drinks on a single occasion), age of first alcohol consumption for both a sip, full drink and drinking to excess, the maximum number of standard drinks they have consumed on one occasion, the proportion of their friends who drink and their intention to try alcohol. These questions were originally adapted from the School Health and Alcohol Harm Reduction Project (SHAHRP) ‘Patterns of Alcohol’ index [52] and trials of the ‘*Preventure’* program [53], and reflect those used in previous *CLIMATE Schools* trials [47,48,51,54]. Other drug use will be measured by questions based on the 2010 National Drug Strategy Household Survey (NDSHS) [45] and the 2011 Australian Secondary Students Alcohol and Drug Survey (ASSAD) [55]. This allows for comparison between use in the current sample and a large scale representative group of Australians. Students will also be asked to rate on a five-point Likert scale, ranging from ‘*very likely’* to ‘*very unlikely’,* how likely it is that they will try alcohol, cannabis and any other drugs in the future.

#### ***Other substance use measures***

Alcohol related harms will be assessed using the 23-item Rutgers Alcohol Problem Index (RAPI) [56]. Students will be asked to rate how many times in the past six months they have experienced harms such as, “*neglected my responsibilities”,* as a consequence of drinking alcohol, on a five point Likert scale ranging from ‘*never’* to ‘*more than 6 times’*. Alcohol related knowledge will be assessed using a 16-item questionnaire originally adapted from the SHAHRP ‘Knowledge of Alcohol’ index [52], and reflect those used in previous trials using the *CLIMATE Schools* programs [47,48,51,54]. Participants’ knowledge about cannabis will be assessed by the 16-item ‘Knowledge about Cannabis’ scale used in previous *CLIMATE Schools* trials [47,48,51,54].

#### ***Mental health measures***

The ‘Kessler 6’ scale (K6) [57] will be used to assess psychological distress in the past month. Two questions from the ‘Kessler 6 plus’ (K6+) scale will also be included to assess the impact of distress on students’ lives. Depression symptoms will be measured using the ‘Patient Health Questionnaire 8’ (PHQ8) [58], while anxiety symptoms will be measured by the ‘Generalised Anxiety Disorder *7*-item scale’ (GAD-7) [59]. The 3-item ‘Mini Social Phobia Inventory’ will screen for social phobia [60]. Quality of life will be measured by the ‘Child Health Utility 9D’ (CHU 9D) [61,62]. The 25-item ‘Strengths and Difficulties Questionnaire’ (SDQ) [63] will be used to assess both positive and negative attributes of the students. Participants’ knowledge about coping with anxiety and depression will be assessed by twelve questions that cover appraisal, reality focused coping and emotion focused coping.

#### ***Personality type***

The 23-item ‘Substance Use Risk Profile Scale’ (SURPS) [64] will be used to assess for variation in personality risk for substance abuse/dependence along four dimensions: sensation seeking, impulsivity, anxiety sensitivity and hopelessness.

#### ***Peer pressure***

The 10-item *‘*Resistive Self-Regulatory Efficacy Scale’ [65,66] will be used to measure participants’ perceived efficacy to resist peer pressure to engage in high risk activities.

#### ***Program evaluation and teacher log books***

Upon completion of the *CLIMATE Schools* programs, students and teachers will be asked to evaluate the program. Students will be asked to indicate how acceptable, appropriate and enjoyable they found the program and to indicate how likely it is they will use the information in their own lives. Teachers will be asked to rate the program; a) overall, b) in comparison to other substance use education programs, and c) in relation to the educational quality of the program. Teachers will also be asked to indicate how easy the program was to implement, how well it held students’ attention, and how likely it is that they would use the program in the future.

#### ***Implementation and treatment fidelity***

All teachers delivering the *CLIMATE Schools* programs will be asked to complete a logbook, indicating which lessons and activities they completed with their class and to write down any adaptations they made to the program. To ensure completion and fidelity of the online part of the program, students are required to view the lessons in full and in order. Students are not able to access the next lesson until they have completed the previous online lesson in full.

#### ***Record of additional substance use and mental health education***

Health and Physical Education teachers across all of the four study groups will be asked to record any substance use and/or mental health education that was delivered to students and/or their parents that was in addition to the *CLIMATE Schools* programs. Specifically teachers will be asked about the length, content and delivery method of this education.

#### ***Statistical analysis***

Single-level analyses; one-way analyses of variance (for normally distributed data), Chi-square (for binominal data), and Mann-Whitney U-test (for non-normally distributed data) will be used to examine baseline equivalence and attrition between groups. Due to the multi-level and hierarchical nature of the data, mixed effects regression will be used to examine intervention by time interaction effects. To account for intracluster variance within States or schools, intervention effects will primarily be examined using hierarchical linear modelling (HLM) for normally distributed data and hierarchical generalized linear modelling using a Poisson distribution for count data. Outcome variables will be centred at post-test to allow for comparisons between groups immediately after the intervention, and growth terms will be analysed to determine the magnitude of the follow-up effects. Analyses will be conducted using the program Stata. If unconditional models reveal that less than 10% of systematic variance exists at the between State or between school level for any outcome variable, HLM will be abandoned and single-level analyses will be used [67]. For these variables, ANCOVAs utilising the SPSS GLM procedure will be conducted to account for any baseline differences that might exist between groups. For multiple comparisons Bonferroni adjustments will be made. Effect sizes, odds ratios and 95% confidence intervals will also be calculated.

## Discussion

The present study protocol presents the design of a cluster RCT to evaluate the effectiveness of the CSC intervention; an integrated universal program to prevent alcohol and cannabis use and symptoms of anxiety and depression among adolescents. The primary aims of the CSC study are to evaluate the effectiveness of the CSC intervention compared to universal prevention programs for substance use and anxiety/depression when delivered alone and compared to school-based Health and Physical Education as usual in relation to; a) reducing the uptake and harmful use of alcohol and other drugs, b) reducing rates of anxiety and depression, and c) increasing mental health and substance use related knowledge.

### Strengths and limitations

The preventative focus of the CSC study on comorbid substance use and mental health symptoms in young people is a key strength of the study. To date, school prevention programs for anxiety, depression and substance use have mostly been designed to target one disorder at a time, rather than targeting all these common disorders. The development and evaluation of a combined prevention approach to mental health and substance use problems is a critical step in acknowledging the comorbidity of these problems, including common antecedents, as well as influencing policy to improve mental health and wellbeing.

The CSC intervention has been designed to overcome many obstacles and limitations of current school-based preventative programs. First, the program has been specifically designed to link with the curriculum, and is thereby potentially practical, acceptable, and scalable to all schools in Australia. Furthermore, implementation issues that have hindered previous school-based programs have been addressed through the use of innovative online delivery methods, which are designed to increase program fidelity and increase student engagement.

Finally, the study has been powered to allow for comparisons between all four of the intervention conditions, both nationally and within each of the three States for effect sizes as low as .15. Such a large sample will allow for the detection of even small differences between groups. This will enable the investigators to establish the efficacy of the combined program over and above the stand-alone programs, as well as normative substance use and mental health education in Physical Health and Education classes as usual.

One limitation of the study is the inclusion of participant self-report measurement for the primary outcomes of substance use and mental health symptoms. The use of self-report measures introduces the possibility of over or under reporting by participants. However, self-report measures have demonstrated excellent discriminant [68] and predictive [69] validity in relation to substance related symptoms [53]. Self-report remains the most practical and popular method of assessment of substance use and mental health in young people within school settings. There are also currently no other feasible alternatives for data collection on substance use and mental health in these settings, as biological or observational measures would not be appropriate for such a large sample in the early stages of substance use initiation and mental health symptoms [70]. In order to maximise the accuracy of students’ self-report data, the investigators will employ the following strategies: visual prompts in the form of standard drink charts, blind administration of any paper and pencil assessments within schools, and a strong emphasis on anonymity and confidentiality, with a special emphasis placed in the fact that schools and parents will not be given access to individual participants’ data.

## Conclusion

Research has highlighted the comorbidity of anxiety, depressive and substance use disorders, yet there are no existing prevention programs which adopt a combined approach to prevent and reduce symptoms associated with these common disorders. Furthermore, while a number of preventative programs for substance use and mental health symptoms are available, many yield minimal effect sizes, are not universally feasible and do not embody effective implementation strategies. The CSC study will be the first trial, internationally, to test a combined prevention model, whilst addressing the aforementioned limitations identified in current preventive programs.

The CSC intervention has the potential to reduce substance use, as well as reduce rates of anxiety and depression at higher levels than either; a) separate substance use and mental health programs and, b) traditional classroom Health and Physical Education. Importantly, this study offers the potential for a paradigm shift, such that future preventive programs and research adopts a combined approach into understanding comorbid substance use and mental health disorders.

### Ethics approval number

Ethics approval has been granted by the UNSW Human Research Ethics Committee (HC13073).

## Endnote

^1^National Health and Medical Research Council of Australia (NHMRC) Targeted Mental Health Grant.

## Competing interests

MT, NN and GA are three of the developers on the *CLIMATE Schools* program in Australia. These programs are distributed not for profit. The other authors declare that they have no competing interests.

## Authors’ contributions

MT, NN, GA, TS and CC are the Chief Investigators on the CSC study NHMRC grant in Australia. LBirrell, ZT, NN and MT are responsible for ethics and clinical trial submission, recruitment of schools, and data collection. SA and NM are responsible for trail co-ordination, school recruitment and data collection in Western Australia. LH is responsible for trail co-ordination, school recruitment and data collection in Queensland. GA, LM and LBrownhill are responsible to modification of the *CLIMATE Schools - Mental Health* module. All authors were involved in study design and will be involved in data analysis and reporting of the study results. All authors read and approved the final manuscript.

## Pre-publication history

The pre-publication history for this paper can be accessed here:

http://www.biomedcentral.com/1471-244X/14/32/prepub
